# Effect of a sanitation intervention on soil-transmitted helminth prevalence and concentration in household soil: A cluster-randomized controlled trial and risk factor analysis

**DOI:** 10.1371/journal.pntd.0007180

**Published:** 2019-02-11

**Authors:** Lauren Steinbaum, John Mboya, Ryan Mahoney, Sammy M. Njenga, Clair Null, Amy J. Pickering

**Affiliations:** 1 Civil and Environmental Engineering, Stanford University, Stanford, California, United States of America; 2 Epidemiology and Biostatistics, University of Georgia, Athens, Georgia, United States of America; 3 Innovations for Poverty Action, Nairobi, Kenya; 4 Innovations for Poverty Action, New Haven, Connecticut, United States of America; 5 Eastern and Southern Africa Center of International Parasite Control, Kenya Medical Research Institute (KEMRI), Nairobi, Kenya; 6 Mathematica, Washington, D.C., United States of America; 7 Civil and Environmental Engineering, Tufts University, Medford, Massachusetts, United States of America; Imperial College London, Faculty of Medicine, School of Public Health, UNITED KINGDOM

## Abstract

Improved sanitation has been associated with a reduced prevalence of soil-transmitted helminth (STH) infection and has been hypothesized to prevent fecal contamination from spreading throughout the household environment. We evaluated the effect of providing households with a pit latrine with a plastic slab and drophole cover, child feces management tools, and associated behavioral messaging on reducing STH eggs in household soil. We collected soil samples from 2107 households (898 control and 1209 improved sanitation intervention households) that were enrolled in the WASH Benefits cluster randomized controlled trial in rural Kenya and performed a post-intervention analysis after two years of intervention exposure. Following a pre-specified analysis plan, we combined all households that received the sanitation intervention into one group for comparison to control households. The prevalence of STH eggs in soil was 18.9% in control households and 17.0% in intervention households. The unadjusted prevalence ratio of total STH eggs in the intervention groups compared to the control group was 0.94 (95% CI: 0.78–1.13). The geometric mean concentration was 0.05 eggs/g dry soil in control households and intervention households. Unadjusted and adjusted models gave similar results. We found use of a shared latrine, presence of a roof over the sampling area, and the number of dogs owned at baseline was associated with an increased prevalence of STH eggs in soil; the presence of a latrine that was at least 2 years old and a latrine with a covered drophole was associated with a reduction in the prevalence of STH eggs in soil. Soil moisture content was also associated with an increased prevalence of STH eggs in soil. Our results indicate that an intervention designed to increase access to improved latrines and child feces management tools may not be enough to impact environmental occurrence of STH in endemic areas where latrine coverage is already high.

## Author summary

Soil is crucial for the lifecycle and transmission of soil-transmitted helminths (STH), a class of parasitic intestinal worms that infect over a billion people globally. Past studies have shown that water, sanitation, and handwashing may be able to reduce STH infection, but the causal pathway is unclear. This study was nested within a large randomized controlled trial evaluating the impact of individual and combined water, handwashing, and sanitation interventions on child health in western Kenya. The sanitation intervention included upgrading pit latrines by installing a plastic slab, as well as delivery and promotion of child safe feces management tools. The sanitation intervention did not reduce STH contamination in household soil, suggesting households with access to improved sanitation may still be exposed to STH in the household environment. Risk factors for the presence of STH soil contamination included sharing toilets, an uncovered latrine pit, latrines <2 years old, owning dogs, shade on the sampling location, and high soil moisture content.

## Introduction

Soil plays a crucial role in the lifecycle of soil transmitted helminths (STH). Eggs need to incubate in soil in a warm, moist environment to become infective. Hookworm species need up to 14 days to become viable and infectious, *Ascaris lumbricoides* eggs need 8 to 37 days, and *Trichuris trichiura* eggs need 20 to 100 days.[1] Once the eggs become infectious, *Ascaris* and *Trichuris* eggs can remain infectious for a few months and hookworm larvae can remain infectious for a few weeks[1]. Two species of STH, *Ascaris lumbricoides* and *Trichuris trichiura*, are transmitted through the ingestion of infective eggs. Both hookworm species, *Ancylostoma duodenale* and *Necator americanus*, infect people through larvae penetrating the skin, but *Ancylostoma duodenale* is also transmitted by ingesting infective larvae[2]. Soil-transmitted helminth (STH) eggs are excreted in the feces of an infected person and are present in the environment in endemic areas. STH eggs have been found in soil in rural households in Poland, southern Thailand, the Philippines, Tanzania, Kenya, and South Africa; urban households in Ethiopia and Jamaica; informal settlements in Brazil and Turkey; urban and rural towns in Nepal; and primary schools in northern Vietnam and South Africa[3–14].

Soil-transmitted helminth infections are endemic in our study area in rural western Kenya. An evaluation of the Kenya national deworming program reported a reduction in the prevalence of overall STH infection from 2012 to 2014[15]. After two years of annual deworming, the prevalence of STH infection in schoolchildren in Kakamega county dropped from 58.3% to 24.8% (calculated from [15]). Much of this reduction was due to a decrease in hookworm infection, which went from 28.8% to 0.1% (calculated from [15]). The prevalence of *Ascaris* and *Trichuris* infection remained at similar levels at approximately 25% prevalence of *Ascaris* and 1% prevalence of *Trichuris*[15].

Water treatment, sanitation, and handwashing (WSH) interventions may help prevent STH infections. Pit latrines with slabs were classified as improved sanitation facilities by the Joint Monitoring Programme for Water Supply and Sanitation (JMP) under the Millennium Development Goals[16]. Pit latrines with a slab may be more likely to be used and easier to clean, which could reduce egg transfer from the latrine to the household. Ventilated pit latrines with slabs may also reduce the spread of fecal contamination by reducing flies in the latrine. Previous laboratory and field studies have found STH eggs on the bodies of and inside the gut of flies,[17–19] which suggests that flies may be able to transfer STH eggs from one location to another. Improved sanitation facilities may reduce STH infection prevalence by making it more likely that feces are safely contained, thereby reducing STH eggs in the environment and the chance of exposure to infectious STH eggs. A meta-analysis of descriptive, cross-sectional studies on sanitation and STH infection showed the availability and use of improved sanitation facilities was associated with reduced odds of STH infection compared to lack of availability or disuse of sanitation facilities[20]. Another meta-analysis indicated that access to any sanitation facility, improved or unimproved, was associated with reduced odds of STH infection[21].

Few studies have examined the causal pathways by which improved sanitation could reduce STH infection in rural low-income countries. Our hypothesized theory of change is that improved sanitation reduces STH in soil, which then reduces the risk of STH exposure and infection to household members. Our study focused on elucidating the importance of this intermediate step in the potential causal pathway. In this study, we evaluate whether an improved household sanitation intervention—incorporating a pit latrine with plastic slab and drophole cover, a plastic child potty, and a metal scoop to safely dispose of feces in the environment—reduced STH eggs in soil outside of rural houses in western Kenya. We also explore household, latrine, and environmental characteristics that were associated with STH eggs in soil among households in the control arm of our study.

## Methods

### Ethics statement

The study procedures were approved by the Stanford Institutional Review Board (Protocol Number 23310) and the Kenya Medical Research Institute (KEMRI) Ethical Review Committee (SSC Number 2271). All respondents gave written, informed consent prior to participating in the survey and gave oral consent collected electronically prior to soil collection. All respondents were adults.

### Study design and enrollment

We enrolled a subset of households in the WASH Benefits study in Kakamega, Bungoma, and Vihiga counties in western Kenya. The WASH Benefits study was a cluster-randomized controlled trial that assessed the impact of individual and combined water, sanitation, handwashing, and nutrition interventions on child health; the main trial outcomes included diarrhea, growth, parasite infections, and cognitive development[22–25]. We describe the effect of the interventions on child STH infections in the discussion section. We collected soil samples from within the double-sized active control arm (non-WASH related household visits), as well as the single sized sanitation arm, and the single sized combined water, sanitation, and hygiene (WSH) arm in the WASH Benefits randomized controlled trial[22]. The trial enrolled 892 households in the sanitation arm, 912 in the WSH arm, and 1919 in the active control arm[26]. Our study focuses on an intermediate outcome not pre-specified in the original trial protocol. Villages were chosen in rural areas that did not have any ongoing WSH or nutrition interventions. Additionally, >80% households had to lack access to piped drinking water. Households with pregnant women were selected within enrolled villages. At the time of data collection, these households had a young child between 21 and 27 months and many had an older child between the age of 3 and 15. Households were assigned to a cluster of households, and randomization of the intervention was performed by cluster[22]. Each cluster included one or two adjoining villages. Children in our study area may have received deworming medicine as part of the national school-based deworming program[27]. Kenya’s national school-based deworming program included annual deworming of the study area that began in 2012[15,27]. We collected data on consumption of deworming medicine within the past 6 months in each household during data collection.

### Interventions

Households enrolled in the sanitation arm received an improved pit latrine with a plastic slab with raised footings and a drop hole cover, a portable child potty made of plastic, and a metal scoop for removing animal and child feces from the compound and placing it in the latrine. The child potty was designed for use by children under 3 years of age, many of whom practice open defecation daily[22,23]. Households enrolled in the combined WSH arm received the sanitation intervention, as well hardware for facilitating treating drinking water with chlorine and handwashing with soap. The water intervention was designed to improve microbial quality and not water quantity. Chlorine dispensers were installed at community water sources and bottles of chlorine were delivered to homes for treating stored drinking water. Dual tippy taps with soapy water and rinse water were installed at the latrine and food preparation areas, and soap was delivered regularly to promote handwashing. Further details of the interventions and WASH Benefits primary results can be found in Null et al (2018)[26]. All households, including the active control arm, received monthly visits from community promoters who measured the mid-upper arm circumference of children enrolled in the trial; in the sanitation and WSH arms they also delivered behavior change messaging related to the interventions. Promotion materials are published at https://osf.io/26r59/. Follow up survey data, including soil samples, were collected approximately two years after the interventions were delivered.

### Field data collection

**Fig 1:**
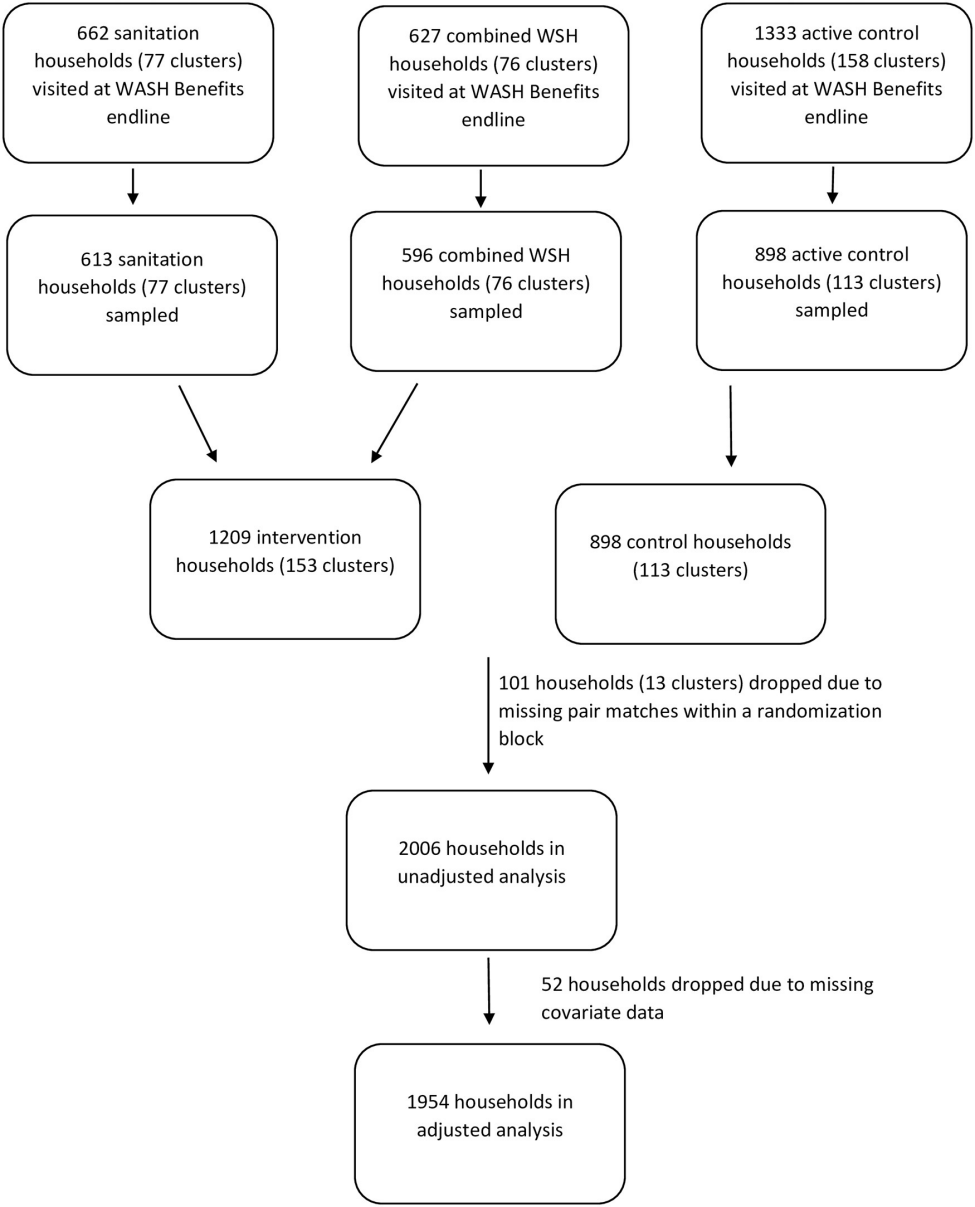
Study flow diagram.

We collected 2107 soil samples from February 2015 to July 2016; 898 from the active control arm, 613 from the sanitation arm, and 596 from the combined WSH arm of the WASH Benefits trial (Fig 1). We collected samples from households in 113 control clusters, 77 sanitation clusters, and 76 WSH clusters. All clusters enrolled in the sanitation and WSH arms of the main trial were enrolled into this study, while a subset (approximately 75%) of clusters was enrolled from the two control groups (grouped together in the trial analyses to form a double-sized control group). Households were enrolled in the WASH Benefits control groups randomly, and our sub-study was only able to visit one control group out of the two control groups within some blocks due to logistical constraints. For each sample, we collected approximately 50 grams of soil within a 900 cm^2^ area that was exposed in front of the primary entrance to the house. Samples were collected by scraping the top layer of soil with a clean metal spade into a sterile Whirlpak bag (118 mL capacity, Nasco, Fort Atkinson, WI). Most (94%) samples were collected within 2 meters of the house entrance. In a few instances (124 out of 2107), we collected a sample over 2 meters away from the house entrance because there was no soil present directly in front of the house entrance. We chose to collect samples at the house entrance because previous work suggested that STH egg presence and concentration was similar at the latrine entrance and house entrance, and we wanted to collect samples from a location that could be standardized across households. The location was also consistent with our theory of change that improved sanitation reduces STH infection through reduced environmental STH contamination in the house environment. Samples were collected throughout the day and the time of collection was noted. A household survey was performed at the same time that the soil samples were collected.

We performed an electronic survey using SurveyCTO, which is a mobile data collection platform based on Open Data Kit (ODK), on Samsung Galaxy tablets. We collected information about soil sampling and the physical conditions around the sampling site, including presence of trash, presence of animal or human feces, presence of visible soil moisture, presence of a roof extending over the sampling location, the presence of sun on the sampling location at the sampling time, and the characteristics of the soil (hard or soft packed, presence of vegetation, and presence of brick or concrete under the soil). We also collected information about the type and number of animals that households kept.

### Laboratory analysis

We analyzed soil samples for the presence of STH eggs following the protocol outlined in Steinbaum et al. (2017)[28]. The protocol has an *Ascaris* recovery efficiency of 73% and has been used to detect *Ascaris* and *Trichuris* in field studies in Kenya and Bangladesh, but its ability to detect hookworm eggs and larvae is unknown. We selected this standardized protocol because *Ascaris* was the most prevalent soil-transmitted helminth in our study area[15]. Samples were transported from the field to the laboratory at room temperature. They were placed in a 4°C refrigerator immediately upon arrival to the laboratory and remained there until processing. We microscopically examined and enumerated the number of fertilized and larvated *Ascaris*, *Trichuris*, and hookworm eggs in each sample, and we incubated all positive samples to determine egg viability. We classified an egg a fertilized egg if it was fertilized, including single-celled and multiple-celled eggs, and it did not contain a larvae. We classified an egg a larvated egg if it contained a larvae before incubation. We classified an egg a viable egg if it contained a larvae after incubation. We also determined the moisture content and soil texture of each sample following the protocol in Steinbaum et al. (2017)[28]. Separate aliquots of each soil sample were used for measuring moisture content through oven drying and for STH egg enumeration.

We incorporated several levels of quality assurance and quality control (QAQC) into the microscopy portion of the laboratory analysis. Soil laboratory technicians counted the number of STH eggs in each sample before and after incubation. A microscopy expert reviewed a subset (14%, 295 out of 2107) of the microscopy slides. Additionally, we took a photo of the first non-larvated and larvated egg of every STH species that we found in every sample prior to incubation. A microscopy expert reviewed every photo and provided feedback to the laboratory technicians. We also included QAQC measures into our laboratory process. Each laboratory technician processed a technical replicate of the soil sample in every other day and a laboratory blank on the alternate days. We defined a technical replicate as an additional 15g aliquot of homogenized soil that was processed using the same laboratory procedure as the original sample aliquot. Our laboratory blank was 5 mL distilled water that was processed using the same procedure as the original sample aliquot. We did not detect contamination in any of our laboratory blanks. The technicians and microscopy expert were blinded to the treatment assignments. The detection limit of the method is 1 egg per 15g wet weight of soil sample.

### Data analysis

We calculated the prevalence of eggs as the binary presence or absence of any eggs in a sample and the concentration of eggs as the number of eggs per gram of dry soil that was processed. We measured viable eggs because they are infectious, and we considered eggs to be viable if they contained a larvae after incubation. Percent viability was calculated as the percent of eggs in each sample that were viable out of the total number of eggs.

Our pre-specified primary analysis (S1 Appendix) compares the presence of eggs in soil between households that received the sanitation intervention (combining WSH and sanitation arms) compared to the control group. The control group had varying levels of sanitation coverage (SI). A subgroup analysis was performed to understand if the combined WSH intervention had a greater reduction in eggs in soil compared to the sanitation intervention. We performed this analysis for the following outcomes: binary presence of all STH eggs, all *Ascaris* eggs, all *Trichuris* eggs, viable STH eggs, viable *Ascaris* eggs, and viable *Trichuris* eggs. We planned to also perform the analysis with the outcome of hookworm eggs or larvae in soil, but we did not perform this analysis because we did not find any hookworm eggs or larvae in our samples. The prevalence of hookworm infection in study children in the control group was low at 2.2% [25]. We used a generalized linear model with a Poisson distribution and log link with the presence of eggs as the dependent variable and the intervention as the independent variable. The model included fixed effects for randomization block and accounted for clustering using Hubert Sandwich Estimator robust standard errors. We ran this model for all outcomes. We modified our model specification from logistic regression, which was indicated in the pre-specified plan, to Poisson regression because of convergence issues.

Our pre-specified secondary analysis (S1 Appendix) compares the concentration of STH in soil between households that received the interventions compared to the control group. We also compared the concentration of STH eggs in soil in the WSH and sanitation interventions. We used a targeted maximum likelihood estimation model with Gaussian distribution and log link to estimate the soil egg count reduction in intervention arms compared to the control arm. We performed this analysis using a continuous outcome of the concentration of all STH eggs, all *Ascaris* eggs, all *Trichuris* eggs, viable STH eggs, viable *Ascaris* eggs, and viable *Trichuris* eggs. We calculated the reduction in the concentration of STH eggs in soil using the geometric mean. To perform this analysis, we log10-transformed the concentration of STH eggs in soil. For non-detect samples, we substituted the concentration of 0 eggs/g dry soil with half of the detection limit (0.5 egg/sample) divided by the grams of dry soil processed per sample, then took the log10 to get a concentration in log10-units of eggs/g dry soil.

We analyzed adjusted models for the primary and secondary analysis. We prescreened covariates and only included those that had an association (p<0.2) with the outcome. We assessed the association of the following covariates: past deworming of a young child (under 3 years), soil texture (sandy loam or clay loam), sunlight exposure on sampling location, a roof over the sampling location, soil moisture content, month of sampling, house materials (concrete floor and iron roof), and household assets (electricity, radio, television, mobile phone, clock, bicycle, motorcycle, stove, number of cows, number of goats, number of dogs, and number of chickens). Month was included as a categorical (factor) variable. Wealth indicator data (house materials and household assets) were collected during baseline data collection for the WASH Benefits study. Minor deviations from our pre-specified analysis plan included removal of several covariates in the adjusted analysis due to a large number of missing values, including past deworming of an older child, rain within the past week, temperature, and humidity. We also included a covariate for the laboratory technician who processed the sample in the adjusted models. We used robust standard errors clustered by randomization block for all of our analysis. Since randomization was performed with pair-matched clusters, 101 observations were dropped that did not have a corresponding pair match. There were also 48 observations dropped from the adjusted analysis due to missing covariate data. We performed the primary and secondary analysis using R version 3.4.2.

Additionally, we followed a pre-specified analysis plan (S1 Appendix) to assess the extent to which household, latrine, and environmental characteristics were associated with the prevalence of STH eggs in household soil in all households that had access to an observable latrine, including factors not expected to be affected by the sanitation intervention. We chose our independent variables based on a few hypotheses. Household factors—number of household members, past deworming of household members, safe child feces management (child feces collected in potty or diaper and thrown in latrine)—may influence initial egg deposition in the household environment. Latrine factors—observed presence of a latrine, self-reported age of latrine, observed presence of concrete or plastic latrine slab, observed presence of drop hole cover, and observed cleanliness of the latrine—may prevent STH egg deposition and transfer in the house environment. Additionally, environmental factors—soil moisture content, soil texture, presence of sun[12] or a roof over the sampling area, past rain, temperature, humidity, and month[1]—may affect the persistence and viability of STH eggs in the environment. Finally, individual wealth indicators—iron roof, concrete floor, electricity, radio, TV, mobile phone, clock, bicycle, radio, stove, number of cows, number of goats, number of dogs, number of poultry—could confound the association between the explanatory variables, i.e. household and latrine factors, and the outcome.

We assessed bivariate associations between these variables and the presence of any STH eggs in soil. Then, we included any variables that had a significant association (p<0.2) with the outcome in a multivariable Poisson regression model with Hubert Sandwich Estimator robust standard errors. We assessed variance inflation factors and excluded correlated variables. Our final model includes use of a shared latrine, presence of a roof over the sample site, presence of a latrine slab, presence of a fully covered drophole, visible stool on the latrine floor, a young child (under 3 years) dewormed within the past 6 months, presence of a latrine that is at least two years old, soil moisture content, and the number of dogs owned at baseline as independent variables. Independent variables in the multivariable model were considered significantly associated with the presence of STH eggs in soil at α = 0.05 (p-value<0.05). We used Stata version 13 to perform this analysis.

Finally, we used logistic regression to assess the association between sample storage time and the presence of STH eggs in soil samples, and to assess the association between individual lab technician and the presence of STH eggs in soil samples.

## Results

### Baseline sanitation coverage

At baseline, 3.3% (30/896) of control households did not have access to a toilet, 77.2% (692/896) had a toilet without a slab (i.e. unimproved latrine), and 15.1% (135/896) had a toilet with a slab (i.e. improved sanitation facilities) that was observed by an enumerator. In control households, respondents reported that 3.3% of young children under 3 years, 46.3% of young children age 3 to 7 years, 83.9% of men, and 85.9% of women in the household always use the latrine. Additional information about baseline characteristics of WASH Benefits households can be found in the primary results manuscript from the WASH Benefits trial.[23]

### Endline household characteristics

Few control households owned pigs (5.7%) and some households owned dogs (18.9%) (Table 1). Overall, we found 77 households in the study kept pigs. Most control households had earth floors (94.4%) defined as mud covering 80–100% of the floor inside the house. Respondents reported that 40.0% of young children under the age of 3 and 41.2% of older children between 3 and 15 years were dewormed within the past month.

**Table 1:** Endline household characteristics in control, sanitation, and WSH households.

	Control	Sanitation	WSH
**Pig ownership**	5.7% (44/767)	4.5% (21/468)	2.5% (12/476)
**Dog ownership**	18.9% (145/767)	19.8% (93/468)	19.0% (91/478)
**Earth floor (80–100% of floor inside house)**	94.4% (846/896)	94.1% (577/613)	94.3% (561/595)
**Young child under 3 years dewormed within past 6 months**	40.0% (359/898)	36.7% (225/613)	37.9% (226/596)
**Older child between 3 and 15 years dewormed within past 6 months**	51.8% (370/714)	52.6% (252/479)	48.5% (227/468)

### Endline WSH coverage in intervention and control groups

Two years after the start of intervention activities, 20.2% of WSH households had detectable free chlorine in stored water at the time of sampling and 20.3% had soap and water available for handwashing (S5 Table). Free chlorine was detected in stored water in 2.0% of control households and 2.0% of sanitation households. Soap and water were present in handwashing areas in 9.1% of control households and 9.0% of sanitation households. In sanitation and WSH households, 79.6% and 83.7% had access to an improved latrine compared to 17.9% of control households. Stool was present on the floor of the latrine in 30.3% of control households, 23.3% of sanitation households, and 23.3% of WSH households. Reported adult open defecation was low with 0.1% of respondents reporting it in the active control arm, 0.2% in the sanitation arm, and 0.2% in the WSH arm. The intervention’s metal scoops were observed in 4.1% of control households, 71.0% of sanitation households, and 60.7% of WSH households, and were reported as the primary tool for child feces removal in 0.1% of control households, 72.9% of sanitation households, and 61.8% of WSH households. Child potties were observed in 3.0% of control households, 84.5% of sanitation households, and 82.7% of WSH households. Young children were reported to have used a potty for more than half of defecation events in the past week in 1.6% of control households, 33.8% of sanitation households, and 28.9% of WSH households. In sanitation and WSH study arms, 35.7% and 34.1% of households reported disposing of child feces in the latrine compared to 9.7% of control households.

### STH egg prevalence and concentration in soil

We found STH eggs in 17.8% of all soil samples (Table 2). *Ascaris* was the most prevalent STH egg (13.0%), followed by *Trichuris* (6.9%). We did not find any hookworm eggs or larvae in any of our samples. The geometric mean concentration of total STH eggs in all samples was 0.05 egg/g dry soil (Table 3). We found the mean of the percent of viable *Ascaris* eggs at each household was 83.5% and the mean of the percent of viable *Trichuris* eggs at each household was 73.2%. The intraclass correlation for the presence of STH eggs by cluster was 0.07 for any STH, 0.05 for *Ascaris*, and 0.04 for *Trichuris*. The presence of any STH eggs before and after incubation was consistent in 76.1% of samples. The presence of eggs in technical replicates was consistent in 88.7% of samples for *Ascaris* and 94.4% for *Trichuris*. We did not find a significant effect of sample storage time on the presence of STH eggs in soil (odds ratio [OR] = 0.97, 95% CI = 0.92–1.02, p = 0.26). We did find a difference in the proportion of eggs positive for any STH by which of our two main laboratory technicians counted the sample (OR = 2.95, 95% CI = 2.33–3.74, p<0.01). None of our blanks were contaminated with STH eggs.

**Table 2:** Prevalence of at least one STH egg detected in the soil sample by intervention arm.

	All (N = 2107)	Control (N = 898)	Sanitation (N = 613)	WSH (N = 596)
**All STH**	17.8%	18.9%	17.5%	16.6%
***Ascaris***	13.0%	13.8%	12.9%	11.7%
***Trichuris***	6.9%	7.6%	6.5%	6.4%
**Hookworm spp**.	0%	0%	0%	0%

**Table 3:** Geometric mean concentration of all STH eggs and viable STH eggs. Non-detect samples were replaced by half the detection limit for each sample.

	All (N = 2096)	Control (N = 894)	Sanitation (N = 610)	WSH (N = 592)
**All STH (eggs per dry g)**	0.052	0.053	0.051	0.050
**All Viable STH (eggs per dry g)**	0.048	0.049	0.047	0.046
***Ascaris* (eggs per dry g)**	0.048	0.049	0.048	0.046
***Trichuris* (eggs per dry g)**	0.040	0.041	0.039	0.040

The mean moisture content of soil samples was 11.0% (SD = 9.8%). The dry weight of processed soil samples ranged from 6.1 g to 15.6 g, with a mean of 13.4 g. The mean temperature in the middle of the compound at the time of sample collection was 27.3°C (SD = 3.3°C) and the mean relative humidity at the time of sample collection was 56.6% (SD = 12.7%). The most common soil textures were sandy loam and clay loam. We found 37.3% (785 out of 2105) of samples were sandy loam and 41.5% (873 out of 2105) were clay loam. Silty loam accounted for 0.9% of samples (18 out of 2105), silty clay loam for 2% (42 out of 2105), silty clay for 3.7% (77 out of 2105), loam for 7.8% (163 out of 2105), clay for 3.1% (65 out of 2105), sandy clay loam for 3.1% (65 out of 2105), and sandy clay for 0.8% (17 out of 2105). Two samples were not tested for soil texture. At the time of collection, 40.7% (860 out of 2017) of samples were collected from areas with hard packed soil and 57.9% (1219 out of 2107) of samples were collected from areas with soft packed soil.

### Effect of the interventions on STH eggs in soil

We did not find an effect of the improved household sanitation intervention on the presence of total STH eggs, *Ascaris* eggs, or *Trichuris* eggs (Table 4). The unadjusted prevalence ratio of total STH eggs in the intervention groups (mean 17.0%) compared to the control group (mean 18.9%) was 0.94 (95% CI: 0.78–1.13), and the unadjusted effect of the intervention on total viable STH eggs was similar (PR = 0.97; 95% CI: 0.76–1.25) (Table 4). When we adjusted for covariates, we still did not find any significant decrease in the prevalence of STH eggs (PR = 0.94; 95% CI: 0.78–1.13) (Table 4, S2 Appendix). In our subgroup analysis, we did not find a significant effect of the sanitation only group or WSH intervention group on the presence of STH eggs in soil (S1 and S3 Tables). When analyzing each species of STH, we found no significant reduction in the prevalence of *Ascaris* eggs (PR = 0.96; 95% CI: 0.76–1.21) or *Trichuris* eggs (PR = 0.81; 95% CI: 0.51–1.16) in the intervention and control groups (Table 4). Additionally, we saw similar results for viable eggs as we did for total STH eggs (Table 4).

**Table 4:** Effect of sanitation intervention (sanitation and WSH combined vs. control) on presence of STH eggs in soil. A prevalence ratio < 1 indicates a reduction of STH eggs.

	All Eggs				Viable Eggs			
	Unadjusted (N = 2006)		Adjusted (N = 1958)		Unadjusted (N = 2006)		Adjusted (N = 1958)	
	Prevalence ratio (95% CI)	p	Prevalence ratio (95% CI)	p	Prevalence ratio (95% CI)	p	Prevalence ratio (95% CI)	p
**Any STH**	0.94 (0.78–1.13)	0.51	0.94 (0.76–1.17)	0.59	0.97 (0.76–1.25)	0.84	0.96 (0.72–1.26)	0.75
***Ascaris***	0.96 (0.76–1.21)	0.76	0.99 (0.76–1.28)	0.91	1.03 (0.77–1.40)	0.83	1.03 (0.74–1.44)	0.86
***Trichuris***	0.81 (0.57–1.16)	0.26	0.86 (0.62–1.19)	0.38	0.71 (0.45–1.13)	0.15	0.74 (0.46–1.21)	0.23

Additionally, the intervention did not significantly reduce the concentration of total STH eggs or the concentration of viable STH eggs in soil (Table 3). We did not detect an intervention effect for total STH, *Ascaris*, or *Trichuris* eggs (Table 5). We saw a slight reduction in viable *Trichuris* eggs in the unadjusted analysis, but the effect was not detected in the adjusted analysis (Table 5, S2 Appendix). In our subgroup analysis, we did not detect a difference between total and viable STH in the sanitation only and control arms or between the WSH and control arm (S2 and S4 Tables). We saw a slight reduction of viable *Trichuris* in the unadjusted analysis of the sanitation intervention, but the effect was not significant in the adjusted analysis (S4 Table).

**Table 5:** Effect of sanitation intervention (sanitation and WSH combined vs control) on concentration of STH eggs in soil. A percent egg count difference > 0 indicates an increase of STH eggs in soil and < 0 indicates a decrease of STH eggs in soil.

	All eggs				Viable eggs			
	Unadjusted (N = 1995)		Adjusted (N = 1947)		Unadjusted (N = 2003)		Adjusted (N = 1955)	
	Percent egg count difference (95% CI)	p	Percent egg count difference (95% CI)	p	Percent egg count difference (95% CI)	p	Percent egg count difference (95% CI)	p
**Any STH**	-2.2% (-5.8%, 1.3%)	0.22	-0.1.2% (-4.6%, 2.2%)	0.49	-1.8% (-5.2%, 1.5%)	0.29	-0.5% (-3.8%, 2.8%)	0.75
***Ascaris***	-1.8% (-5.2%, 1.7%)	0.31	-0.4% (-3.7%, 3.0%)	0.83	-1.1% (-4.3%, 2.2%)	0.53	0.2% (-3.0%, 3.4%)	0.90
***Trichuris***	-1.4% (-3.3%, 0.5%)	0.16	-0.5% (-2.0%, 1.1%)	0.53	-1.5% (-2.6%, 0.4%)	0.01	-0.7% (-1.7%, 0.4%)	0.22

We performed the analysis excluding the 77 households that kept pigs, and the results were consistent with the reported results.

We included use of a shared latrine, presence of a roof over the sampling site, presence of a latrine slab, presence of a completely covered drophole, presence of stool on the latrine floor, past deworming of a young child (under 3 years old), use of a latrine that is at least 2 years old, percent soil moisture content, and the number of dogs owned at baseline in the multivariable model (S6 Table, S1 Text, Table 6). Use of a shared latrine was associated with a 39% increase in the prevalence of STH eggs in soil (Table 6, PR = 1.39, CI = (1.13, 1.72), p < 0.01) and the presence of a roof fully covering the sample site was associated with a 27% increase in the prevalence of STH eggs in soil (Table 6, PR = 1.27, CI = (1.05, 1.53), p = 0.01). Increasing soil moisture content was associated with an increased prevalence of STH eggs in soil. Each additional dog owned by the household at baseline was associated with an 11% increase in the presence of STH eggs in soil (Table 6, PR = 1.11, (1.05, 1.18), p < 0.01). Having the latrine drophole completely covered was associated with a 34% reduction in the prevalence of STH eggs in soil (Table 6, PR = 0.66, CI = (0.48, 0.91), p = 0.01), and having a latrine that was at least 2 years old was associated with a 17% reduction (Table 6, PR = 0.83, CI = (0.70, 0.99), p = 0.03).

**Table 6:** Multivariable Poisson regression model of factors associated with presence of STH in soil in all households (N = 1899). Households without latrine access are excluded from the model to allow for inclusion of variables related to latrine characteristics.

	Prevalence Ratio	95% Confidence Interval	p
Use of shared latrine in households with access to a latrine	1.39	(1.13, 1.72)	<0.01
Presence of roof over sampling site	1.27	(1.05, 1.53)	0.01
Presence of latrine slab in households with an observable latrine	0.93	(0.77, 1.13)	0.49
Drophole fully covered in households with an observable latrine	0.66	(0.48, 0.91)	0.01
Stool present on latrine floor in households with an observable latrine	1.15	(0.92, 1.43)	0.22
Young child (under 3 years) dewormed up to 6 months before sampling	0.85	(0.68, 1.05)	0.13
Latrine at least 2 years old	0.83	(0.70, 0.99)	0.03
Percent soil moisture content	1.02	(1.01, 1.02)	<0.01
Number of dogs owned at baseline	1.11	(1.05, 1.18)	<0.01

## Discussion

Among all study households, we found 17.8% (N = 376/2107) of households had STH eggs in soil from the house entrance. *Ascaris* eggs were most common, which is consistent with STH infection prevalence in the area. The geometric mean concentration of STH in soil from all households was 0.05 eggs/g dry soil. An average of 79.9% of these eggs were viable, indicating that they pose a human health risk.

Our results indicate the sanitation intervention was not effective in reducing the prevalence of STH eggs in household soil. We also did not find an added benefit of the WSH intervention compared to the sanitation intervention delivered alone on reducing STH eggs in soil. It is useful to interpret our findings together with the observed effects of the WSH and single sanitation interventions on actual child STH infection prevalence levels in the same trial. Infection prevalence with *Ascaris* was significantly lower among children in the WSH arm compared to the control arm, while there was no statistically significant reduction among children in the single sanitation arm[25]. The lack of a health impact in the single sanitation arm is consistent with the lack of effect of the improved sanitation intervention on soil STH levels. The reduction in STH infection prevalence observed in the WSH arm could be due to the water treatment or handwashing with soap components of the WSH intervention interrupting other environmental STH transmission pathways (e.g. ingestion through drinking water or food contaminated by hands).

Our results are consistent with a cross-sectional study in Tanzania by Exley et al. that found no difference in STH eggs in composite soil samples in urban and rural compounds with different levels of sanitation on the JMP sanitation ladder[29]. Additionally, a study in Brazil found there was no correlation between the concentration of *Ascaris* eggs in composite household soil samples in households with varying levels of sanitation quality[30]. However, some households had flush toilets that emptied into the community and children practiced open defecation, which potentially spread *Ascaris* eggs. In contrast to previous studies, our intervention also included child feces management tools that were thought to potentially reduce STH eggs in soil originating from child feces. However, uptake of the tools was incomplete at endline.

A potential explanation for the null effect of our intervention is that transitioning from access to unimproved sanitation to access to improved sanitation may not have a measurable impact on STH eggs in household soil; transitioning a population from prevalent open defecation to using even unimproved sanitation may be more likely to affect the presence and concentration of STH eggs in soil. In our study, many respondents in control households reported that their household had access to a latrine that was in use (98.1%, 881/898). According to the JMP definition, the main difference between an unimproved and an improved latrine is the presence of a plastic or concrete slab[16]. A benefit of a plastic or concrete slab is that it is easier to clean, and our study found that receiving our improved sanitation intervention was associated with reduced stool on the floor of the latrine. The presence of stool on the latrine floor makes it more likely that fecal contamination could be spread from the latrine to other areas in the house.

Our multivariable regression results indicated that a shared latrine was associated with an increased prevalence of STH eggs in soil, which is consistent with previous cross-sectional studies that have shown an association between shared latrines and increased helminth infection[31]. Mahfouz et al. (1997) indicated that increased child STH infection (OR = 1.95, 95% CI = 1.38–2.75) was associated with living in households where latrines were shared[32]. A potential explanation is that shared latrines are less clean and may have a greater likelihood of containing STH eggs because they are used by a larger number of people than a private household latrine. People who use a shared latrine may track contaminated feces from the latrine to their households, where the eggs incubate and become infective. Heijnen et al. (2015) found that shared latrines in India were more likely to be dysfunctional, less clean, and to have flies and feces. Additionally, they found that people who have access to a shared latrine were more likely to practice open defecation,[33] which would reduce the effectiveness of the latrine in preventing environmental contamination.

Our multivariate modeling also revealed several additional household and environmental factors to be associated with the presence of STH eggs in soil. Presence of a roof over the sampling area was also associated with increased prevalence of STH eggs in soil. Sampling soil in sunny areas has been associated with lower egg counts in soil in previous studies[12,14]. Higher moisture content was associated with STH presence, which may be due to the persistence of helminth eggs in moist soil under temperate conditions[1]. The number of dogs owned by a household at baseline was associated with increased STH eggs in soil. Studies have shown dogs can transmit *Trichuris trichiura* and *Ascaris lumbricoides* in their stool [34,35] and suggest that they may also carry infectious eggs on their fur[35]. However, there was no significant association with dog ownership at endline. Additionally, presence of a latrine that was at least 2 years old and a latrine with a covered drophole were associated with reduced prevalence of STH eggs in soil. Since STH eggs are persistent in the environment, a household with a new latrine could have residual STH contamination from prior to latrine installation. Latrines with a covered drophole may prevent flies from entering the latrine and depositing feces and STH eggs throughout the house environment.

One limitation of our study was that we do not have precise measures of compound defecation practices. Measurement of the uptake of the sanitation intervention focused on the physical presence of an improved latrine or respondent-reported measures of usual latrine use and defecation behaviors within the past week for some compound members. Also, an increased sample size would have decreased the minimum detectable effect. Another limitation is that the soil analysis method has been optimized for *Ascaris* recovery. The recovery efficiency of the method using *Trichuris* and hookworm eggs and larvae is unknown, and field tests in Kenya and Bangladesh did not detect any hookworm. Another potential study limitation is that pigs can carry *Ascaris* with eggs that are microscopically indistinguishable from humans; however, less than 6% of households in our study that reported animal ownership kept pigs. Also, excluding households that kept pigs from the analysis did not change results. Additionally, despite thorough training and QA/QC throughout the study, the laboratory technician effect was a significant covariate in our adjusted analyses. However, for most of the study, the two laboratory technicians worked in different field laboratories that received samples from different study areas, either Kakamega and Vihiga or Bungoma. Future studies should try to mitigate this effect through additional training and by hiring additional laboratory technicians. Finally, our analysis of STH risk factors in the control group is cross-sectional and may be susceptible to confounding. The context of the study should be considered when generalizing the results; our study was carried out during a national school-based deworming campaign in an area that already had high access to sanitation facilities.

Our study indicates an improved household sanitation intervention did not reduce the presence or concentration of STH eggs in household soil within our soil sampling area in our study area in rural Kenya. Our study compared households with an improved sanitation intervention with those in a control group who mostly had access to unimproved sanitation facilities. Kenya is one of the few countries in sub-Saharan Africa where elimination of STH as a public health problem may be possible because of the transmission intensity, capacity of funding partners, and political and financial logistics and infrastructure[36]. As home environments have been identified as an important consideration for elimination of STH infection in Kenya,[36] more work should be done to evaluate drivers of household soil STH contamination and their potential impact on STH infection and elimination.

## Supporting information

S1 Appendix Pre-specified analysis plan. (DOCX)

S2 Appendix Covariates included in Tables 4 and 5. (DOCX)

S1 Table Effect of sanitation intervention (sanitation vs. control) on presence of STH eggs in soil. A prevalence ratio < 1 indicates a reduction of STH eggs.
(DOCX)

S2 Table Effect of sanitation intervention (sanitation vs control) on concentration of STH eggs in soil. A percent egg count difference < 0 indicates a decreased concentration of STH eggs in soil.
(DOCX)

S3 Table Effect of WSH intervention (WSH vs. control) on presence of STH eggs in soil. A prevalence ratio < 1 indicates a reduction of STH eggs.
(DOCX)

S4 Table Effect of WSH intervention (WSH vs control) on concentration of STH eggs in soil. A percent egg count difference < 0 indicates a decreased concentration of STH eggs in soil.
(DOCX)

S5 Table Indicators of intervention uptake in study arms 2 years after intervention delivery. (DOCX)

S6 Table Bivariate associations between household, latrine, and environmental characteristics and the presence of STH in soil using Poisson regression in all households. We considered p<0.2 as significant for the multivariate analysis. N = 1899.
(DOCX)

S1 Text Removal of correlated variables in multivariable analysis. (DOCX)
