# Supplementary material for: Effect of a sanitation intervention on soil-transmitted helminth prevalence and concentration in household soil: A cluster-randomized controlled trial and risk factor analysis
Source: PLoS Negl Trop Dis. 2019 Feb 11;13(2):e0007180. doi: 10.1371/journal.pntd.0007180 (PMC6386409; doi:10.1371/journal.pntd.0007180)
Supplement: S1 Appendix — (DOCX) [file pntd.0007180.s001.docx]

**S1 Appendix. Pre-specified analysis plan**

We pre-specified our analysis plan and revised it until all co-authors were in agreement. We wanted to ensure that our assessment of the intervention effect was pre-specified so that we could not influence our results. Our analysis plan was finalized on March 10, 2017.

**Research Objectives:**

Past work has shown that water, sanitation, and hygiene interventions can reduce the prevalence of STH infection ^1,2^. A potential explanation is that WASH interventions reduce fecal contamination in the environment, which then reduces exposure to STH (particularly in households where a household member or visitor is infected). It has been hypothesized that improved sanitation reduces household contamination with STH, thereby reducing exposure to STH. Our combined WASH intervention also included chlorine to improve drinking water and a handwashing device to promote handwashing. Chlorine may delay embryonation ^3^, and therefore infectivity, of STH eggs in water, but it cannot remove or inactivate eggs. Handwashing may reduce ingestion of eggs from hand-to-mouth contact. The WASH Benefits study had low uptake of the water intervention (~20%) and handwashing intervention (~20%), so the sanitation intervention is likely driving results in the combined WASH intervention.

The main objective of this work is to determine whether a sanitation or combined-WASH intervention ^4^ reduces STH contamination of household soil. Another objective is to understand the factors that may increase or decrease the risk of STH contamination in household soil. Household factors—number of household members, past deworming of household members, child feces management—may influence initial egg deposition in the household environment. Latrine factors—presence of a shared or household latrine, age of latrine, presence of a slab or drop hole cover, and cleanliness of the latrine—may impact the prevention of STH egg transmission from the latrine through the household. Additionally, environmental factors—soil moisture content, soil texture, soil exposure to sun ^5^ and rain, temperature, humidity, and season ^6^—may impact the persistence of STH eggs in the environment. By filling these knowledge gaps, we can further focus and design interventions to reduce STH contamination in the environment.

**Primary Outcomes:**

- Binary prevalence of viable STH eggs(*Ascaris, Trichuris,* hookworm) in soil for any STH and each individual STH
- Binary prevalence of STH eggs in soil
  - Viable STH is useful for assessing potential risk of infection, but total eggs might be a better indicator of fecal contamination in the household.

**Secondary Outcomes:**

- Concentration of viable STH eggs (*Ascaris, Trichuris,* hookworm) in soil for any STH and STH specific contamination
- Concentration of STH in soil eggs

**Covariates to increase precision:**

- Proportion of household members previously dewormed (calculated from reported deworming and household size)
- Soil texture
- Full sunlight exposure on sampling location (or roof covering sampling area)
- Past rain (within a week)
- Soil moisture content
- Temperature
- Humidity
- Month of sampling

**Minimum Detectable Effect Size:**

In our original sample size calculations we decided that soil samples will be collected from approximately 2200 households (~733 per arm). We planned to collect the same number of samples in the control, sanitation, and combined WASH arms of the study. Assuming a baseline prevalence of 19%, 80% power, an intra-class correlation coefficient of 0.2, we estimated the minimum detectable difference as 9 percentage points.

During data collection, we decided to increase sampling in the control group. We went from a target of 1/3 of samples in each arm to ½ of samples in the control group and ¼ of samples in each intervention group. Our final dataset includes uptake survey and soil microscopy data for 2107 households, which is less than we originally planned. The difference is likely due to high attrition for the initial uptake survey visit and missing soil microscopy data from the lab.

**Analysis:**

**H1:** Access to improved sanitation will reduce STH eggs in household soil.

**H2:** Household and latrine characteristics affect the odds of STH in household soil.

Our primary analysis will compare the prevalence and concentration of STH eggs in soil between households that received an improved sanitation intervention (combining WASH and sanitation arms) compared to the control group. A subgroup analysis will explore if the combined WASH intervention had a greater reduction in STH in soil compared to a sanitation intervention. Our analyses will only be able to detect an effect if it is close to the effect size in our sample size calculations. This study is a randomized controlled trial stratified by geographic location, so it is expected that there is no confounding. We will run unadjusted and adjusted models, which should increase the precision of our estimates.

We will use a generalized linear model with a binomial distribution and log link to determine whether the intervention reduced the prevalence of STH in soil (**H1**). We will use a Poisson distribution for the adjusted model. We will use a targeted maximum likelihood estimation model with Gaussian distribution to determine whether the intervention reduced the concentration of STH in soil. We will calculate the reduction of the geomean of STH eggs in soil. We will use robust standard errors clustered by randomized matched cluster pairs.

We will use a generalized linear model with Poisson distribution to determine which household and latrine characteristics affect the risk of STH contamination in household soil (**H2**). We will perform this analysis only on households in the active control arm. We will focus on household, latrine, and environmental characteristics that may influence the presence of and spread of contamination through the household environment. We will assess univariate associations between many factors. Then, we will choose the predictors that have a significance of p<0.2 and evaluate them in a multivariate model. Our final model will comprise predictors that are significant at the p<0.05 level in the multivariate model. We will assess the following variables:

- Proportion of household members previously dewormed (calculated from reported deworming and household size)
- Child defecation outside of the latrine
- Disposal of child feces in latrine
- Number of household members
- Number of household members in school
- How long the latrine has been present
- Presence of a latrine
- Presence of shared latrine
- Presence of a latrine with slab
- Presence of a latrine with drop hole cover
- Visible human feces on latrine slab
- Presence of water and any type of soap at handwashing station
- Soil texture
- Full sunlight exposure on sampling location (or roof covering sampling area)
- Past rain (within a week)
- Soil moisture content
- Temperature
- Humidity
- Month of sampling

**References**

(1) Strunz, E. C.; Addiss, D. G.; Stocks, M. E.; Ogden, S.; Utzinger, J.; Freeman, M. C. Water, Sanitation, Hygiene, and Soil-Transmitted Helminth Infection: A Systematic Review and Meta-Analysis. *PLoS Med.* **2014**, *11* (3), e1001620 DOI: 10.1371/journal.pmed.1001620.

(2) Ziegelbauer, K.; Speich, B.; Mäusezahl, D.; Bos, R.; Keiser, J.; Utzinger, J. Effect of Sanitation on Soil-Transmitted Helminth Infection: Systematic Review and Meta-Analysis. *PLoS Med.* **2012**, *9* (1), e1001162 DOI: 10.1371/journal.pmed.1001162.

(3) Oh, K. S.; Kim, G. T.; Ahn, K. S.; Shin, S. S. Effects of Disinfectants on Larval Development of Ascaris Suum Eggs. *Korean J. Parasitol.* **2016**, *54* (1), 103–107 DOI: 10.3347/kjp.2016.54.1.103.

(4) Arnold, B. F.; Null, C.; Luby, S. P.; Unicomb, L.; Stewart, C. P.; Dewey, K. G.; Ahmed, T.; Ashraf, S.; Christensen, G.; Clasen, T.; Dentz, H. N.; Fernald, L. C. H.; Haque, R.; Hubbard, A. E.; Kariger, P.; Leontsini, E.; Lin, A.; Njenga, S. M.; Pickering, A. J.; Ram, P. K.; Tofail, F.; Winch, P. J.; Colford, J. M. Cluster-Randomised Controlled Trials of Individual and Combined Water, Sanitation, Hygiene and Nutritional Interventions in Rural Bangladesh and Kenya: The WASH Benefits Study Design and Rationale. *BMJ Open* **2013**, *3* (8), e003476 DOI: 10.1136/bmjopen-2013-003476.

(5) Baker, S. M.; Ensink, J. H. J. Helminth Transmission in Simple Pit Latrines. *Trans. R. Soc. Trop. Med. Hyg.* **2012**, *106* (11), 709–710 DOI: 10.1016/j.trstmh.2012.08.002.

(6) Brooker, S.; Clements, A.; Bundy, D. Global Epidemiology, Ecology and Control of Soil-Transmitted Helminth Infections. *Adv. Parasitol.* **2006**, *62* (5), 221–261 DOI: 10.1016/S0065-308X(05)62007-6.
