# Supplementary material for: Effect of a sanitation intervention on soil-transmitted helminth prevalence and concentration in household soil: A cluster-randomized controlled trial and risk factor analysis
Source: PLoS Negl Trop Dis. 2019 Feb 11;13(2):e0007180. doi: 10.1371/journal.pntd.0007180 (PMC6386409; doi:10.1371/journal.pntd.0007180)
Supplement: S2 Appendix — (DOCX) [file pntd.0007180.s002.docx]

**S2 Appendix. Covariates included in Table 4 and 5**

**Covariates Included in Table 4 (Control vs Intervention):**

- Adjusted, any STH prevalence: soil moisture content, young child dewormed within 6 months, sun on sampling area, month, baseline cows, baseline dogs, baseline poultry, technician
- Adjusted, *Ascaris* prevalence*:* soil moisture content, young child dewormed within past 6 months, sun on sampling area, baseline roof, baseline electricity, baseline clock, baseline cows, baseline poultry, technician
- Adjusted, *Trichuris* prevalence: soil moisture content, month, baseline roof, baseline electricity, baseline radio, baseline mobile phone, baseline bicycle, baseline motorcycle, baseline stove, baseline dogs, technician
- Adjusted, viable STH prevalence: moisture content, young child dewormed within past 6 months, clay loam soil, sun on sampling area, month, baseline roof, baseline cows, baseline poultry, technician
- Adjusted, viable *Ascaris* prevalence: soil moisture content, young child dewormed within 6 months, sun on sampling area, month, baseline roof, baseline electricity, baseline cows, baseline poultry, technician
- Adjusted, viable *Trichuris* prevalence: soil moisture content, month, presence of dogs at baseline, technician

**Covariates Included in Table 5 (Control vs Intervention):**

- Adjusted, any STH concentration: soil moisture content, clay loam soil, sun on sampling area, month, baseline roof, baseline radio, baseline clock, technician
- Adjusted, *Ascaris* concentration: soil moisture content, clay loam soil, sun on sampling area, month, baseline roof, baseline floor, baseline electricity, baseline radio, baseline television, baseline mobile phone, baseline clock, baseline cows, baseline poultry, technician
- Adjusted, *Trichuris* concentration: soil moisture content, sandy loam soil, clay loam soil, sun on sampling area, month, baseline roof, baseline electricity, baseline bicycle, baseline motorcycle, baseline stove, baseline dogs, technician
- Adjusted, viable STH concentration: soil moisture content, clay loam soil, sun on sampling area, month, baseline roof, baseline floor, baseline electricity, baseline radio, baseline cows, baseline poultry, technician
- Adjusted, viable *Ascaris* concentration: soil moisture content, clay loam soil, sun on sampling area, month, baseline roof, baseline floor, baseline electricity, baseline radio, baseline mobile phone, baseline cows, baseline poultry, technician
- Adjusted, viable *Trichuris* concentration: soil moisture content, sandy loam soil, clay loam soil, sun on sampling area, month, baseline roof, baseline clock, baseline stove, baseline cows, baseline dogs, technician
