# Supplementary material for: Effect of a sanitation intervention on soil-transmitted helminth prevalence and concentration in household soil: A cluster-randomized controlled trial and risk factor analysis
Source: PLoS Negl Trop Dis. 2019 Feb 11;13(2):e0007180. doi: 10.1371/journal.pntd.0007180 (PMC6386409; doi:10.1371/journal.pntd.0007180)
Supplement: S1 Table — A prevalence ratio < 1 indicates a reduction of STH eggs. (DOCX) [file pntd.0007180.s003.docx]

**S1 Table. Effect of sanitation intervention (sanitation vs. control) on presence of STH eggs in soil.** A prevalence ratio < 1 indicates a reduction of STH eggs.

|  | **All Eggs** | | | | **Viable Eggs** | | | |
| --- | --- | --- | --- | --- | --- | --- | --- | --- |
|  | **Unadjusted (N=1407)** | | **Adjusted (N=1375)** | | **Unadjusted (N=1407)** | | **Adjusted (N=1375)** | |
|  | **Prevalence ratio (95% CI)** | **p** | **Prevalence ratio (95% CI)** | **p** | **Prevalence ratio (95% CI)** | **p** | **Prevalence ratio (95% CI)** | **p** |
| **Any STH** | 0.94 (0.76-1.17) | 0.59 | 0.94 (0.73-1.21) | 0.64 | 1.00 (0.76-1.32) | 0.98 | 0.99 (0.73-1.36) | 0.97 |
|  |  |  |  |  |  |  |  |  |
| ***Ascaris*** | 0.95 (0.73-1.22) | 0.67 | 1.01 (0.76-1.36) | 0.92 | 1.01 (0.74-1.39) | 0.93 | 1.07 (0.74-1.55) | 0.72 |
|  |  |  |  |  |  |  |  |  |
| ***Trichuris*** | 0.87 (0.56-1.35) | 0.53 | 0.90 (0.58-1.39) | 0.66 | 0.79 (0.42-1.48) | 0.46 | 0.71 (0.37-1.39) | 0.32 |
|  |  |  |  |  |  |  |  |  |

**Covariates Included in Model:**

- Adjusted, any STH prevalence: soil moisture content, young child dewormed within past 6 months, clay loam soil, sun on sampling area, month, baseline bicycles, baseline cows, baseline goats, baseline poultry, technician
- Adjusted, *Ascaris* prevalence: soil moisture content, young child dewormed within past 6 months, sun on sampling area, month, baseline roof, baseline electricity, baseline goats, baseline poultry, technician
- Adjusted, *Trichuris* prevalence: soil moisture content, clay loam soil, sun on sampling area, baseline roof, baseline electricity, baseline radio, baseline mobile phone, baseline bicycle, baseline dogs, technician, month
- Adjusted, viable STH prevalence: soil moisture content, clay loam soil, sun on sampling area, month, baseline roof, baseline cows, baseline goats, baseline poultry, technician
- Adjusted, viable *Ascaris* prevalence: soil moisture content, sun on sampling area, month, baseline roof, baseline electricity, baseline cows, baseline goats, baseline poultry, technician
- Adjusted, viable *Trichuris* prevalence: soil moisture content, clay loam soil, month, baseline roof, baseline dogs, technician
