# Supplementary material for: Effect of a sanitation intervention on soil-transmitted helminth prevalence and concentration in household soil: A cluster-randomized controlled trial and risk factor analysis
Source: PLoS Negl Trop Dis. 2019 Feb 11;13(2):e0007180. doi: 10.1371/journal.pntd.0007180 (PMC6386409; doi:10.1371/journal.pntd.0007180)
Supplement: S2 Table — A percent egg count difference < 0 indicates a decreased concentration of STH eggs in soil. (DOCX) [file pntd.0007180.s004.docx]

**S2 Table. Effect of sanitation intervention (sanitation vs control) on concentration of STH eggs in soil.** A percent egg count difference < 0 indicates a decreased concentration of STH eggs in soil.

|  | **All eggs** | | | | **Viable eggs** | | | |
| --- | --- | --- | --- | --- | --- | --- | --- | --- |
|  | **Unadjusted (N=1407)** | | **Adjusted (N=1375)** | | **Unadjusted (N=1407)** | | **Adjusted (N=1375)** | |
|  | **Percent egg count difference**  **(95% CI)** | **p** | **Percent egg count difference**  **(95% CI)** | **p** | **Percent egg count difference**  **(95% CI)** | **p** | **Percent egg count difference**  **(95% CI)** | **p** |
| **Any STH** | -1.8% (-6.4%, 2.7%) | 0.43 | -0.9% (-4.6%, 2.9%) | 0.64 | -1.1% (-5.4%, 3.1%) | 0.60 | -0.7% (-4.5%, 3.0%) | 0.70 |
| ***Ascaris*** | -1.6% (-5.8%, 2.7%) | 0.48 | -0.3% (-4.0%, 3.5%) | 0.88 | -1.0% (-5.0%, 3.0%) | 0.63 | -0.3% (-3.9%, 3.3%) | 0.88 |
| ***Trichuris*** | 0.8% (-2.1%, 3.8%) | 0.57 | -0.5% (-2.2% 1.1%) | 0.53 | -1.6% (-2.9%, -0.2%) | 0.03 | -0.5% (-1.6%, 0.6%) | 0.38 |

**Covariates Included in Model:**

- Adjusted, any STH concentration: soil moisture content, clay loam soil, sun on sampling area, month, baseline roof, baseline mobile phone, baseline clock, baseline goats, technician
- Adjusted, *Ascaris* concentration: soil moisture content, clay loam soil, sun on sampling area, month, baseline roof, baseline electricity, baseline radio, baseline mobile phone, baseline clock, baseline cows, baseline goats, technician
- Adjusted, *Trichuris* concentration: soil moisture content, sandy loam soil, clay loam soil, sun on sampling area, month, baseline roof, baseline electricity, baseline radio, baseline bicycle, baseline dogs, technician
- Adjusted, viable STH concentration: soil moisture content, clay loam soil, sun on sampling area, month, baseline roof, baseline floor, baseline cows, technician
- Adjusted, viable *Ascaris* concentration: soil moisture content, clay loam soil, sun on sampling area, month, baseline roof, baseline floor, baseline electricity, baseline mobile phone, baseline cows, technician
- Adjusted, viable *Trichuris* concentration: soil moisture content, sandy loam soil, clay loam soil, sun on sampling area, month, baseline stove, baseline dogs, technician
