# Supplementary material for: Effect of a sanitation intervention on soil-transmitted helminth prevalence and concentration in household soil: A cluster-randomized controlled trial and risk factor analysis
Source: PLoS Negl Trop Dis. 2019 Feb 11;13(2):e0007180. doi: 10.1371/journal.pntd.0007180 (PMC6386409; doi:10.1371/journal.pntd.0007180)
Supplement: S3 Table — A prevalence ratio < 1 indicates a reduction of STH eggs. (DOCX) [file pntd.0007180.s005.docx]

**S3 Table. Effect of WSH intervention (WSH vs. control) on presence of STH eggs in soil.** A prevalence ratio < 1 indicates a reduction of STH eggs.

|  | **All Eggs** | | | | **Viable Eggs** | | | |
| --- | --- | --- | --- | --- | --- | --- | --- | --- |
|  | **Unadjusted (N=1382)** | | **Adjusted (N=1349)** | | **Unadjusted (N=1382)** | | **Adjusted (N=1349)** | |
|  | **Prevalence ratio (95% CI)** | **p** | **Prevalence ratio (95% CI)** | **p** | **Prevalence ratio (95% CI)** | **p** | **Prevalence ratio (95% CI)** | **p** |
| **Any STH** | 0.94 (0.73-1.19) | 0.59 | 0.92 (0.71-1.20) | 0.54 | 0.91 (0.65-1.26) | 0.56 | 0.90 (0.64-1.26) | 0.55 |
|  |  |  |  |  |  |  |  |  |
| ***Ascaris*** | 0.98 (0.72-1.32) | 0.89 | 0.98 (0.71-1.35) | 0.89 | 1.02 (0.70-1.49) | 0.91 | 0.99 (0.67-1.46) | 0.95 |
|  |  |  |  |  |  |  |  |  |
| ***Trichuris*** | 0.77 (0.49-1.19) | 0.23 | 0.98 (0.62-1.54) | 0.91 | 0.61 (0.35-1.07) | 0.08 | 0.64 (0.34-1.21) | 0.17 |

**Covariates Included in Model:**

- Adjusted, any STH prevalence: soil moisture content, young child dewormed within past 6 months, sun on sampling site, baseline electricity, baseline cows, technician
- Adjusted, *Ascaris* prevalence: soil moisture content, young child dewormed within past 6 months, sun on sampling area, baseline roof, baseline electricity, baseline clock, baseline cows, baseline poultry, technician
- Adjusted, *Trichuris* prevalence: soil moisture content, baseline roof, baseline television, baseline mobile phone, baseline dogs, technician, month
- Adjusted, viable STH prevalence: soil moisture content, young child dewormed within past 6 months, sun on sampling site, month, baseline roof, baseline electricity, baseline bicycle, baseline cows, baseline dogs, baseline poultry, technician
- Adjusted, viable *Ascaris* prevalence: soil moisture content, young child dewormed within past 6 months, sun on sampling area, baseline roof, baseline electricity, baseline bicycle, baseline cows, baseline poultry, technician
- Adjusted, viable *Trichuris* prevalence: soil moisture content, sun on sampling area, baseline dogs, technician
