# Supplementary material for: Effect of a sanitation intervention on soil-transmitted helminth prevalence and concentration in household soil: A cluster-randomized controlled trial and risk factor analysis
Source: PLoS Negl Trop Dis. 2019 Feb 11;13(2):e0007180. doi: 10.1371/journal.pntd.0007180 (PMC6386409; doi:10.1371/journal.pntd.0007180)
Supplement: S4 Table — A percent egg count difference < 0 indicates a decreased concentration of STH eggs in soil. (DOCX) [file pntd.0007180.s006.docx]

**S4 Table. Effect of WSH intervention (WSH vs control) on concentration of STH eggs in soil**. A percent egg count difference < 0 indicates a decreased concentration of STH eggs in soil.

|  | **All eggs** | | | | **Viable eggs** | | | |
| --- | --- | --- | --- | --- | --- | --- | --- | --- |
|  | **Unadjusted (N = 1374)** | | **Adjusted (N = 1341)** | | **Unadjusted (N = 1379)** | | **Adjusted (N = 1346)** | |
|  | **Percent egg count difference**  **(95% CI)** | **p** | **Percent egg count difference**  **(95% CI)** | **p** | **Percent egg count difference**  **(95% CI)** | **p** | **Percent egg count difference**  **(95% CI)** | **p** |
| **Any STH** | -2.8% (-7.2%, 1.7%) | 0.23 | -1.6% (-6.1%, 2.9%) | 0.50 | -2.3% (-6.1%, 1.6%) | 0.25 | -0.4% (-4.3%, 3.5%) | 0.83 |
| ***Ascaris*** | -2.4% (-6.4%, 1.5%) | 0.23 | -1.0% (-4.9%, 2.9%) | 0.60 | -1.4% (-5.1%, 2.3%) | 0.46 | 0.5% (-3.3%, 4.2%) | 0.81 |
| ***Trichuris*** | -0.8% (-3.2%, 1.5%) | 0.49 | -0.1% (-2.3%, 2.1%) | 0.94 | -1.4% (-3.0%, 0.1%) | 0.68 | -0.7% (-2.2%, 0.7%) | 0.32 |

**Covariates Included in Model:**

- Adjusted, any STH concentration: soil moisture content, deworming of young child within past 6 months, sandy loam soil, clay loam soil, sun on sampling site, baseline electricity, baseline cows, baseline poultry, technician, month
- Adjusted, *Ascaris* concentration: soil moisture content, young child dewormed within past 6 months, sandy loam soil, clay loam soil, sun on sampling area, baseline floor, baseline electricity, baseline cows, baseline poultry, technician, month
- Adjusted, *Trichuris* concentration: soil moisture content, sandy loam soil, clay loam soil, sun on sampling area, month, baseline roof, baseline dogs, technician
- Adjusted, viable STH concentration: soil moisture content, sandy loam soil, clay loam soil, sun on sampling area, month, baseline electricity, baseline dogs, baseline poultry, technician
- Adjusted, viable *Ascaris* concentration: soil moisture content, clay loam soil, sun on sampling area, month, baseline floors, baseline electricity, baseline cows, baseline poultry, technician
- Adjusted, viable *Trichuris* concentration: soil moisture content, sandy loam soil, clay loam soil, sun on sampling area, month, baseline clock, baseline bicycle, baseline cows, baseline dogs, technician
