# Supplementary material for: Effect of a sanitation intervention on soil-transmitted helminth prevalence and concentration in household soil: A cluster-randomized controlled trial and risk factor analysis
Source: PLoS Negl Trop Dis. 2019 Feb 11;13(2):e0007180. doi: 10.1371/journal.pntd.0007180 (PMC6386409; doi:10.1371/journal.pntd.0007180)
Supplement: S5 Table — (DOCX) [file pntd.0007180.s007.docx]

**S5 Table. Indicators of intervention uptake in study arms 2 years after intervention delivery**

| **Intervention uptake measurement** | **Control [% (n/N)]** | **Sanitation [% (n/N)]** | **WSH**  **[% (n/N)]** |
| --- | --- | --- | --- |
| Detectable free chlorine in drinking water | 2.0% (17/845) | 2.0% (11/555) | 20.2% (106/526) |
| Handwashing station has soap and water | 9.1% (82/898) | 9.0% (55/613) | 20.3% (121/596) |
| Access to improved latrine | 17.9% (153/856) | 79.6% (475/597) | 83.7% (483/577) |
| Respondent primarily practices open defecation | 0.1% (1/860) | 0.2% (1/559) | 0.2% (1/532) |
| Stool visible on latrine floor | 30.3% (255/841) | 23.3% (137/589) | 23.3% (133/572) |
| Kipupu scoop observed in household | 4.1%  (37/898) | 71.0%  (435/613) | 60.7%  (362/596) |
| Primarily use kipupu scoop for feces removal | 0.1% (1/896) | 72.9% (446/612) | 61.8% (365/591) |
| Child potty observed in household | 3.0%  (27/898) | 84.5%  (518/613) | 82.7%  (493/596) |
| Use of child potty for more than half of defecation events in the past week | 1.6% (14/898) | 33.8% (207/613) | 28.9% (172/596) |
| Child feces safely disposed | 9.7% (79/819) | 35.7% (199/557) | 34.1% (180/528) |
