# Supplementary material for: Effect of a sanitation intervention on soil-transmitted helminth prevalence and concentration in household soil: A cluster-randomized controlled trial and risk factor analysis
Source: PLoS Negl Trop Dis. 2019 Feb 11;13(2):e0007180. doi: 10.1371/journal.pntd.0007180 (PMC6386409; doi:10.1371/journal.pntd.0007180)
Supplement: S6 Table — We considered p<0.2 as significant for the multivariate analysis. N = 1899. (DOCX) [file pntd.0007180.s008.docx]

**S6 Table. Bivariate associations between household, latrine, and environmental characteristics and the presence of STH in soil using Poisson regression in all households.** We considered p<0.2 as significant for the multivariate analysis. N = 1899.

|  | **Prevalence Ratio**  **(95% Confidence Interval)** | **p** |
| --- | --- | --- |
| **Use of shared latrine in households reporting access to a latrine** | 1.35 (1.09, 1.66) | 0.005 |
| **Presence of water and soap for handwashing** | 1.04 (0.77, 1.42) | 0.787 |
| **Presence of sun on sampling site** | 0.69 (0.58, 0.82) | <0.001 |
| **Presence of roof over sampling site (fully or partially covered)** | 1.32 (1.09, 1.59) | 0.004 |
| **Presence of latrine slab in households with an observable latrine** | 0.79 (0.67, 0.93) | 0.006 |
| **Presence of stool on latrine floor in households with an observable latrine** | 1.24 (0.99, 1.054) | 0.057 |
| **Drophole fully covered in households with an observable latrine** | 0.55 (0.41, 0.75) | <0.001 |
| **Latrine at least 2 years old** | 0.79 (0.66, 0.94) | 0.008 |
| **Presence of rain the week before sampling** | 1.20 (0.88, 1.64) | 0.241 |
| **Young child (under 3 years) dewormed up to 6 months before sampling** | 0.85 (0.69, 1.04) | 0.119 |
| **Older child (3 to 15 years) dewormed up to 6 months before sampling in households with an older child** | 1.00 (0.77, 1.30) | 0.990 |
| **Safe child feces management-child defecates in potty or diaper and feces are thrown in latrine** | 1.02 (0.84, 1.25) | 0.818 |
| **Sandy loam** | 1.06 (0.88, 1.23) | 0.558 |
| **Clay loam** | 1.09 (0.93, 1.29) | 0.280 |
| **Dry season** | 1.08 (0.80, 1.46) | 0.595 |
| **Rainy season** | 0.92 (0.68, 1.24) | 0.595 |
| **Percent soil moisture content** | 1.02 (1.01, 1.03) | <0.001 |
| **Latrine age (months)** | 1.00 (0.99, 1.00) | 0.072 |
| **Temperature (°C)** | 0.97 (0.94, 1.00) | 0.058 |
| **Relative humidity** | 1.01 (1.00, 1.02) | 0.018 |
| **Number of household members** | 1.00 (0.96, 1.04) | 0.982 |
| **Number of households sharing latrine** | 1.08 (1.01, 1.15) | 0.028 |
| **Iron roof at baseline** | 0.85 (0.68, 1.08) | 0.188 |
| **Concrete floor at baseline** | 0.82 (0.53, 1.27) | 0.380 |
| **Electricity at baseline** | 0.91 (0.63, 1.32) | 0.611 |
| **Radio at baseline** | 0.97 (0.79, 1.19) | 0.766 |
| **Television at baseline** | 1.00 (0.73, 1.38) | 0.987 |
| **Mobile phone at baseline** | 1.07 (0.85, 1.36) | 0.534 |
| **Clock at baseline** | 0.93 (0.71, 1.23) | 0.633 |
| **Bicycle at baseline** | 1.12 (0.92, 1.36) | 0.268 |
| **Motorcycle at baseline** | 1.05 (0.76, 1.47) | 0.755 |
| **Stove at baseline** | 1.07 (0.90, 1.27) | 0.438 |
| **Number of cows at baseline** | 0.98 (0.92, 1.05) | 0.660 |
| **Number of goats at baseline** | 0.97 (0.88, 1.07) | 0.547 |
| **Number of dogs at baseline** | 1.09 (1.01, 1.17) | 0.019 |
| **Number of poultry at baseline** | 0.99 (0.98, 1.01) | 0.281 |
