# Supplementary material for: Effect of a sanitation intervention on soil-transmitted helminth prevalence and concentration in household soil: A cluster-randomized controlled trial and risk factor analysis
Source: PLoS Negl Trop Dis. 2019 Feb 11;13(2):e0007180. doi: 10.1371/journal.pntd.0007180 (PMC6386409; doi:10.1371/journal.pntd.0007180)
Supplement: S1 Text — (DOCX) [file pntd.0007180.s009.docx]

**S6 Text. Removal of correlated variables in multivariable analysis**

After performing an initial multivariable Poisson regression, we removed correlated variables based on the variance inflation factor values. Temperature, relative humidity, and soil moisture content were correlated. We decided to use soil moisture content because it is most relevant for our outcome of interest. We decided to use a binary variable for shared use of a latrine, instead of the number of households sharing a latrine, because it is more easily interpretable. We also included a dummy variable for latrines that were at least 2 years old, instead of the age of the latrine in months, in the multivariable model because it is more easily interpretable. The presence of an iron roof at baseline, which was one of our wealth indicators, is similar to the presence of a roof over the sampling area. We decided to include the presence of a roof over the sampling area in our final, multivariable model because it is more relevant for the outcome of interest. We dropped the variable measuring sun on the sampling area because it is correlated with a roof over the sampling area, and the presence of a roof over the sampling area is a more objective measure. In the multivariable model, all variables have a variance inflation factor less than 2.5.
